# A Rare Case of a High-Grade Thymic Squamous Cell Carcinoma Presenting as Cardiac Tamponade

**DOI:** 10.7759/cureus.9366

**Published:** 2020-07-24

**Authors:** Maitreyee Rai, Alan Keogh

**Affiliations:** 1 Internal Medicine, Crozer Chester Medical Center, Upland, USA; 2 Hematology and Oncology, Crozer Chester Medical Center, Upland, USA

**Keywords:** thymic carcinoma, squamous cell thymic carcinoma, pericardial effusion, cardiac tamponade, thymic tumors, immunohistochemistry of squamous cell thymic carcinoma, high-grade thymic squamous cell carcinoma

## Abstract

Thymic tumors (for example, thymomas, thymic carcinomas, and thymic neuroendocrine tumors) are rare tumors. Thymic carcinomas are aggressive thymic epithelial neoplasms with a poor prognosis. Cardiac tamponade as a presenting complaint of malignant thymic carcinoma is rare.

A 64-year-old woman presented to the emergency department with complaints of progressive exertional dyspnea and chest discomfort. On physical examination, she had diminished breath sounds at the left lung base. The chest x-ray showed a mediastinal widening, significant cardiomegaly, and pleural effusion. CT scan of the chest revealed a dominant mediastinal mass, left-sided pleural effusion, and pericardial effusion. Transthoracic echocardiogram showed 3 cm circumferential pericardial effusion, with evidence of cardiac tamponade. An emergent pericardiocentesis and thoracentesis were done. A core needle biopsy of the mediastinal mass revealed a high-grade non-keratinizing squamous cell thymic carcinoma. Immunohistochemistry staining was positive for pan-cytokeratin, high molecular weight cytokeratin, CK 5/6, E-cadherin, p63, epithelial membrane antigen (EMA), and BerEp4. The patient had repeated hospital admissions due to recurrent malignant pericardial effusion and left pleural effusion. The patient was planned for radiation and chemotherapy with oncology.

In our review of literature, the primary squamous cell thymic carcinoma presenting initially as a cardiac tamponade was found to be a rare event. Early diagnosis and treatment are of utmost importance given the aggressive clinical course culminating in to poor outcome.

## Introduction

Thymic neoplasms are exceedingly rare tumors with an estimated incidence of less than 1% of all the adult cancers. Thymic epithelial tumors (TETs) comprise thymomas and thymic carcinomas (TCs) [1]. TC is an exceedingly rare cause of an anterior mediastinal mass. They differ from the more commonly known thymomas in their cellular atypia and a more aggressive clinical course [2]. The initial presentation is largely secondary to the compression of the surrounding mediastinal structures. The most common presenting complaints are cough, chest pain, phrenic nerve palsy, or superior vena cava syndrome. Cardiac tamponade as a presenting complaint of malignant TC is rare. We present a case of a 64-year-old with a cardiac tamponade of unknown origin and found to have high-grade malignant TC (Poster: Maitreyee Rai MD, Alan Keogh MD. A Rare Case of Malignant Thymic Carcinoma Presenting as Recurrent Pericardial Effusion and Tamponade. American College of Physicians Internal Medicine Meeting; May 1, 2020).

## Case presentation

The patient is a 64-year-old woman with a past medical history of sarcoidosis and hypertension, who presented to the emergency department (ED) with complaints of progressive exertional dyspnea and chest discomfort going on for one week. In the ED, her temperature was 97.7 degrees Fahrenheit, blood pressure was 168/109 mmHg, heart rate was 115/minute, respiratory rate was 25/minute, and oxygen saturation was 96% on room air. On general physical examination, she was alert and oriented to time, place, and person, in no acute distress. No scleral icterus or conjunctival pallor was noted. Cardiac examination was significant for tachycardia and distant S1 and S2 heart sounds. She had diminished breath sounds at the left lung base, and no wheezes, crackles, or rhonchi were heard. The abdomen was soft and non-tender with normoactive bowel sounds. Lower extremity had no edema or calf tenderness. The initial laboratory studies are shown in Table 1.

**Table 1:** Results of lab studies PCR, polymerase chain reaction

Laboratory Investigations	
White blood cell (WBC), 4.8-10.8 × 10^3^/µL	8.6
Hemoglobin, 11.6-15.0 g/dL	10.3
Hematocrit, 37.0%-47.0%	31.0
Mean corpuscular volume (MCV), 80.0-98.0 fL	80.9
Mean corpuscular hemoglobin (MCH), 27.0-31.0 pg	26.9
Mean corpuscular hemoglobin concentration (MCHC), 31.0-37.0 g/dL	33.3
Red cell distribution width (RDW), 11.4%-14.7%	14.7
Platelet (PLT), 145-400 × 10^3^/µL	359
Sodium (Na), 135-146 mmol/L	137
Potassium (K), 3.5-5.1 mmol/L	3.6
Chloride (Cl), 96-106 mmol/L	102
Bicarbonate (CO_2_), 24-32 mmol/L	23
Blood urea nitrogen (BUN), 10-20 mg/dL	19
Creatinine (Cr), 0.6-1.1 mg/dL	0.92
Bilirubin, total, 0.3-1.0 mg/dL	1.0
Alkaline phosphatase (ALP), 30-120 U/L	104
Aspartate aminotransferase (AST), 5-27 U/L	31
Alanine transaminase (ALT), 7-52 U/L	32
Troponin, <0.04 ng/mL	0.01
B type natriuretic peptide (BNP), <100 pg/mL	53
Lactic acid, mmol/L	1.8
Procalcitonin, ng/mL	<0.05
Urine analysis	
pH, 5.0-8.0	6.0
Color, clarity	Yellow, clear
Glucose (negative), mg/dL	Negative
Bilirubin (negative), mg/dL	Small
Urobilinogen (negative), mg/dL	1.0
Nitrates (negative)	Negative
Blood (negative), /microliter	Small
Red blood cell (RBC), 0-2/HPF	3-10
WBC, 0-2/HPF	6-10
Leukocyte esterase	Negative
Bacteria	Trace
Influenza A and B PCR	Negative

**Figure 1:**
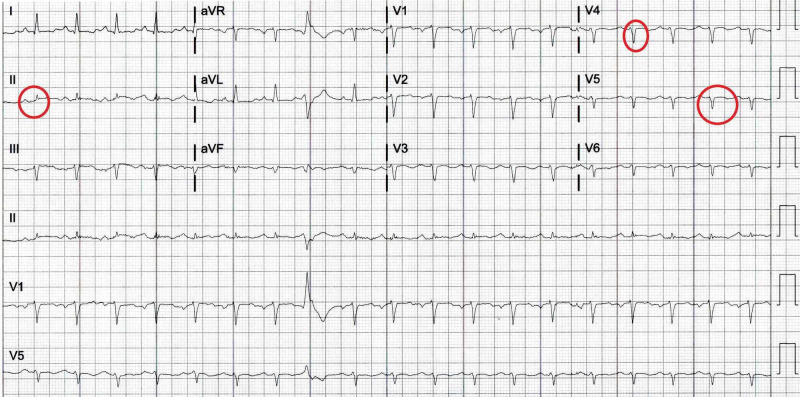
Electrocardiogram (EKG) recording shows low voltage complexes (red encircled).

An electrocardiogram (EKG) showed sinus tachycardia and low voltage QRS complexes (Figure 1).

**Figure 2:**
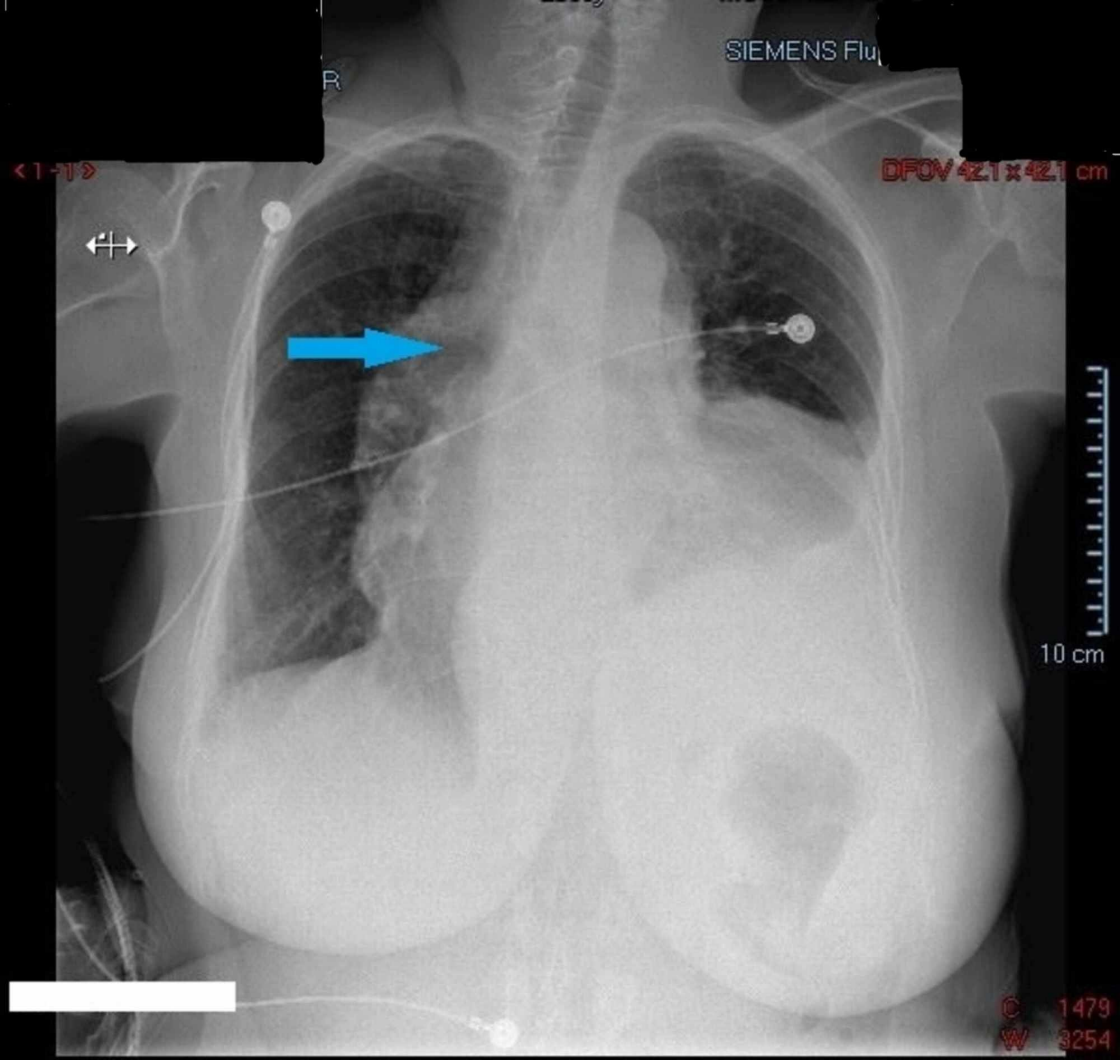
Chest x-ray shows widened mediastinum (blue arrow).

A chest x-ray was done that showed a significantly widened mediastinum, cardiomegaly, left lung lower lobe pleural effusion, compressive atelectasis and/or pneumonia, and clear right lung (Figure 2).

**Figure 3:**
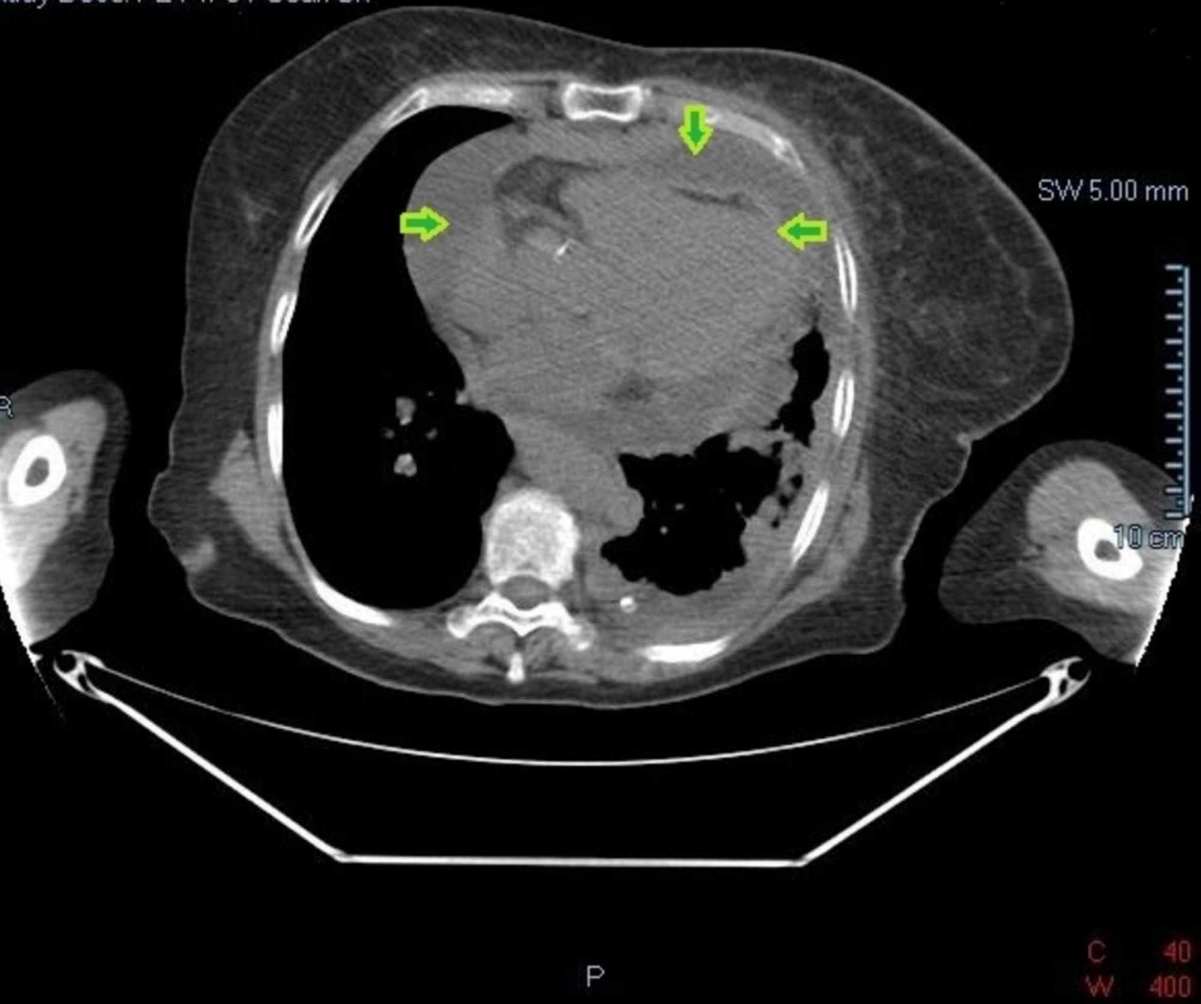
CT chest shows moderate sized pericardial effusion (green arrows).

CT scan of the chest revealed a dominant mediastinal mass with pathological appearing mediastinal lymphadenopathy, left-sided pleural effusion, and pericardial effusion (Figure 3).

**Figure 4:**
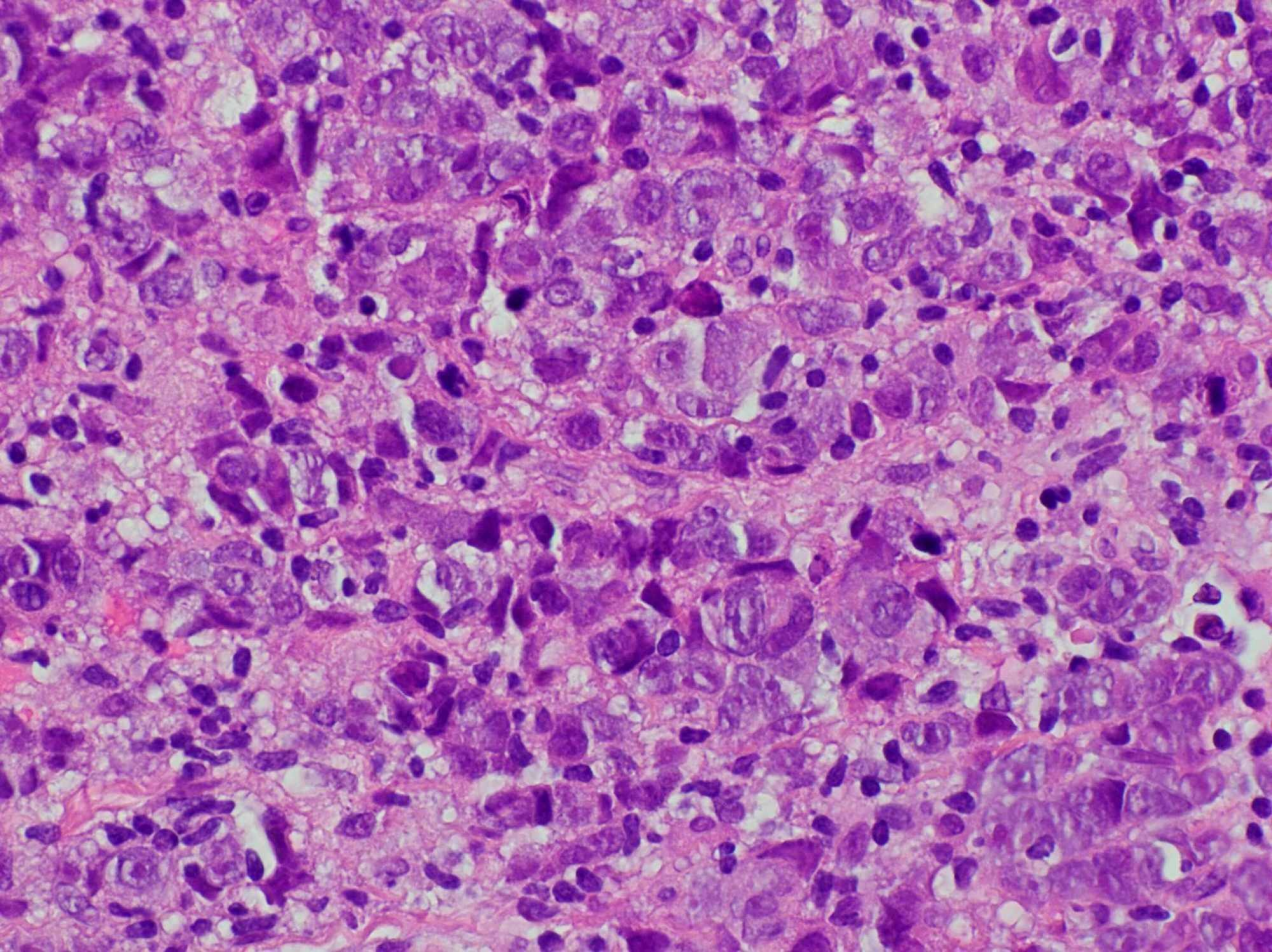
Mediastinal mass core biopsy pathology: hematoxylin and eosin stain. The figure shows polygonal tumor cells of varying size with vesicular nuclei, and abundant eosinophilic glassy cytoplasm.

**Figure 5:**
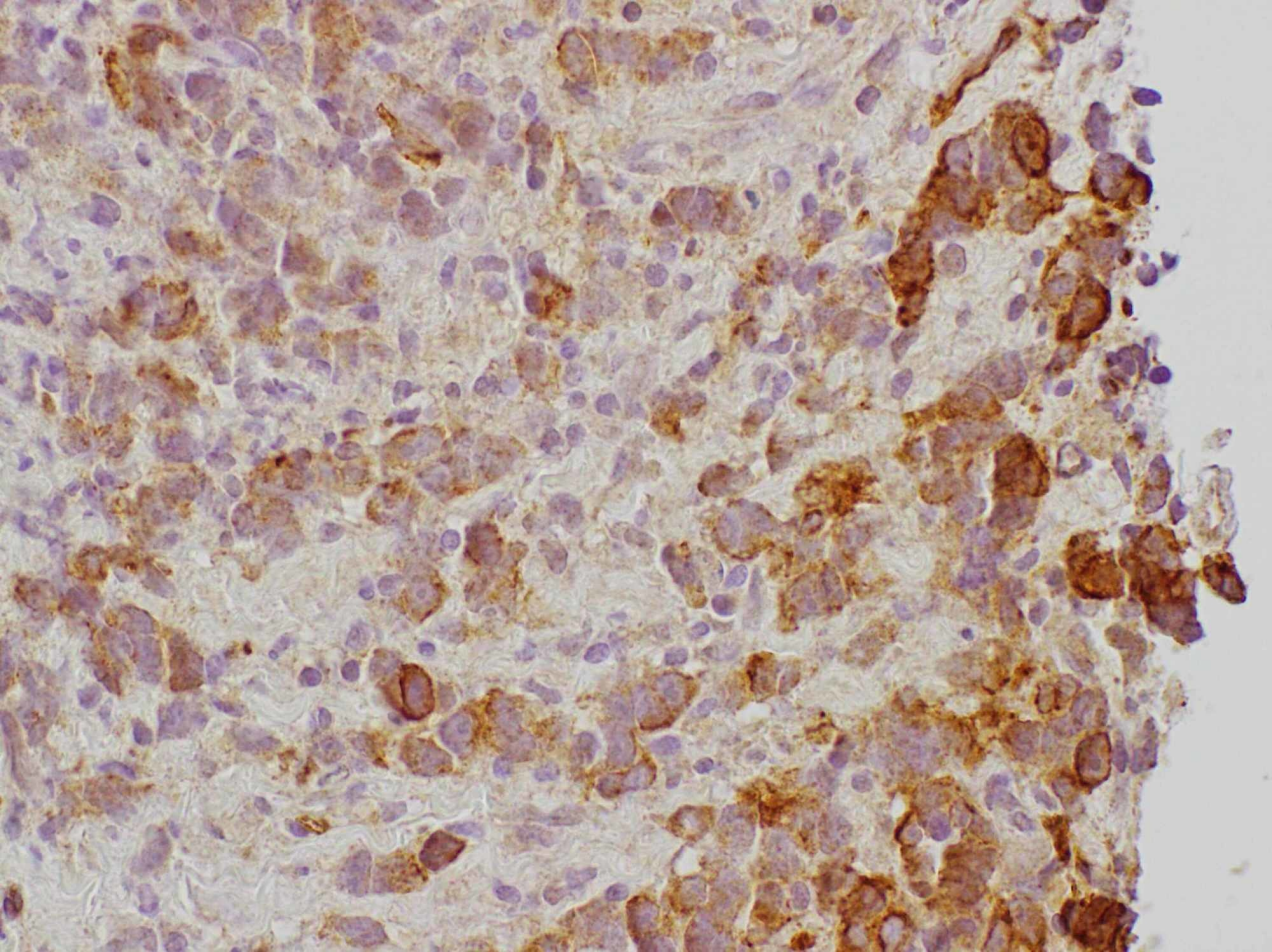
Mediastinal mass core biopsy: pan-cytokeratin stain.

**Figure 6:**
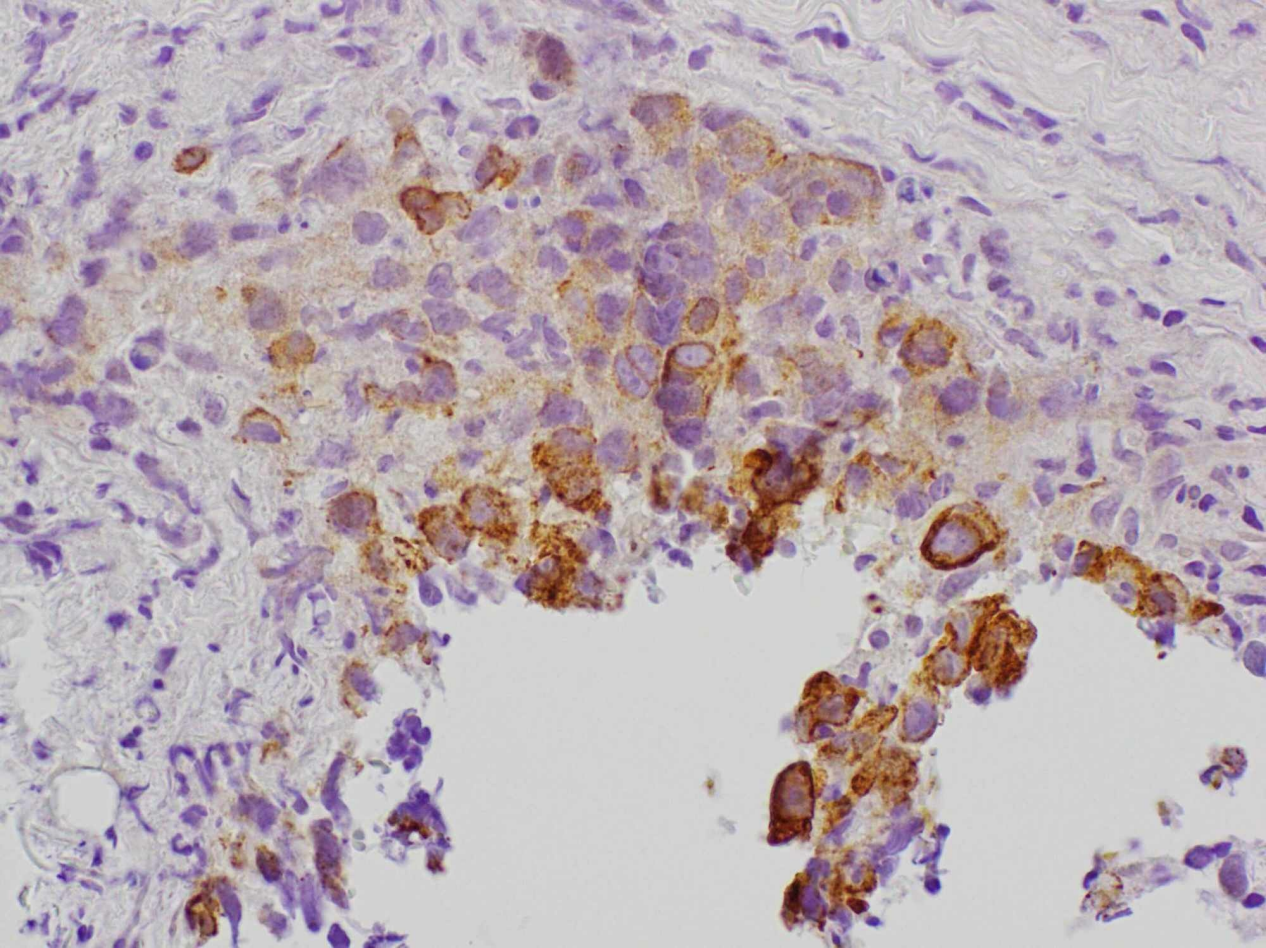
Mediastinal mass core biopsy: high molecular weight keratin stain.

**Figure 7:**
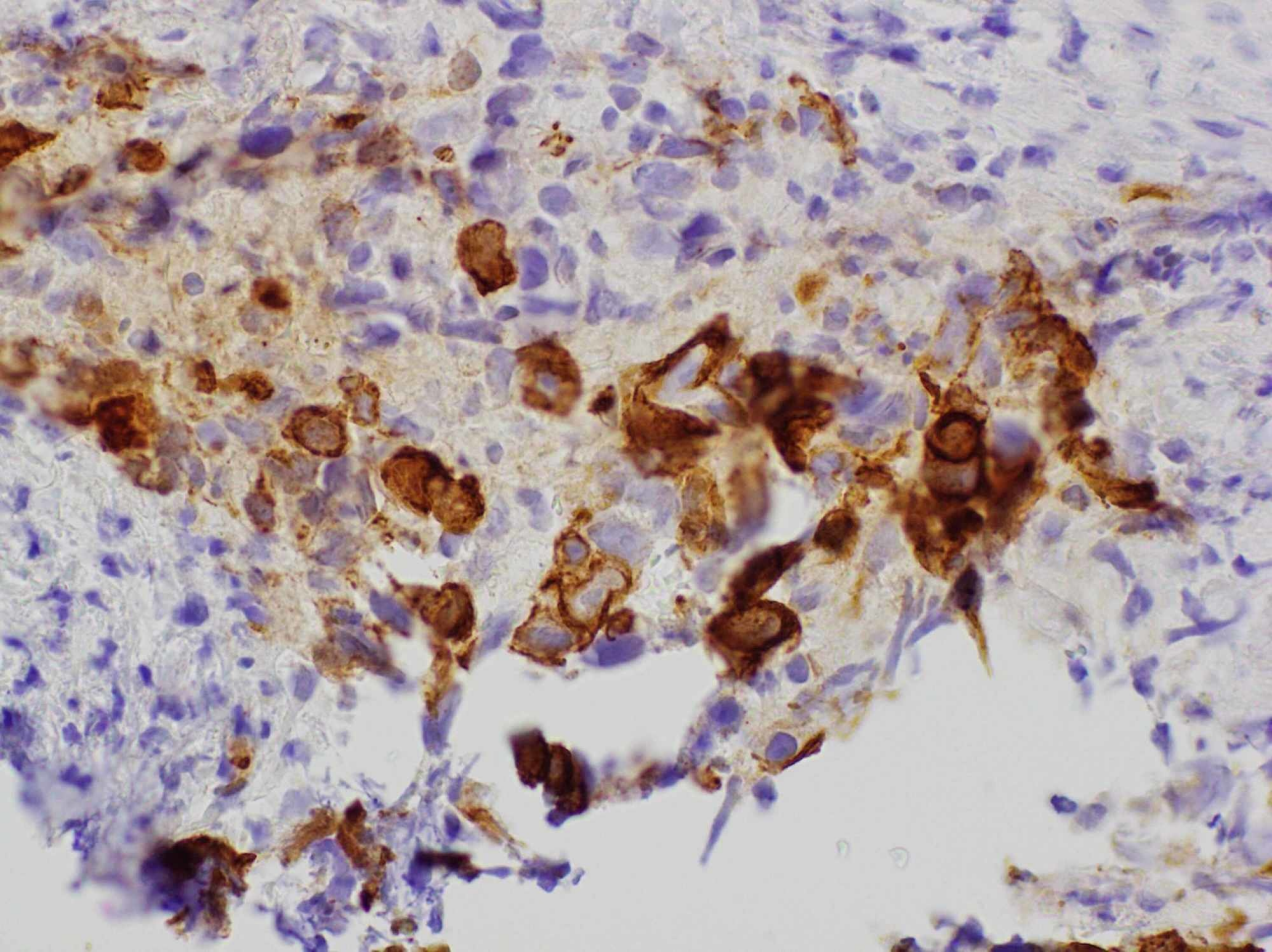
Mediastinal mass core biopsy: cytokeratin CK 5/6 stain.

**Figure 8:**
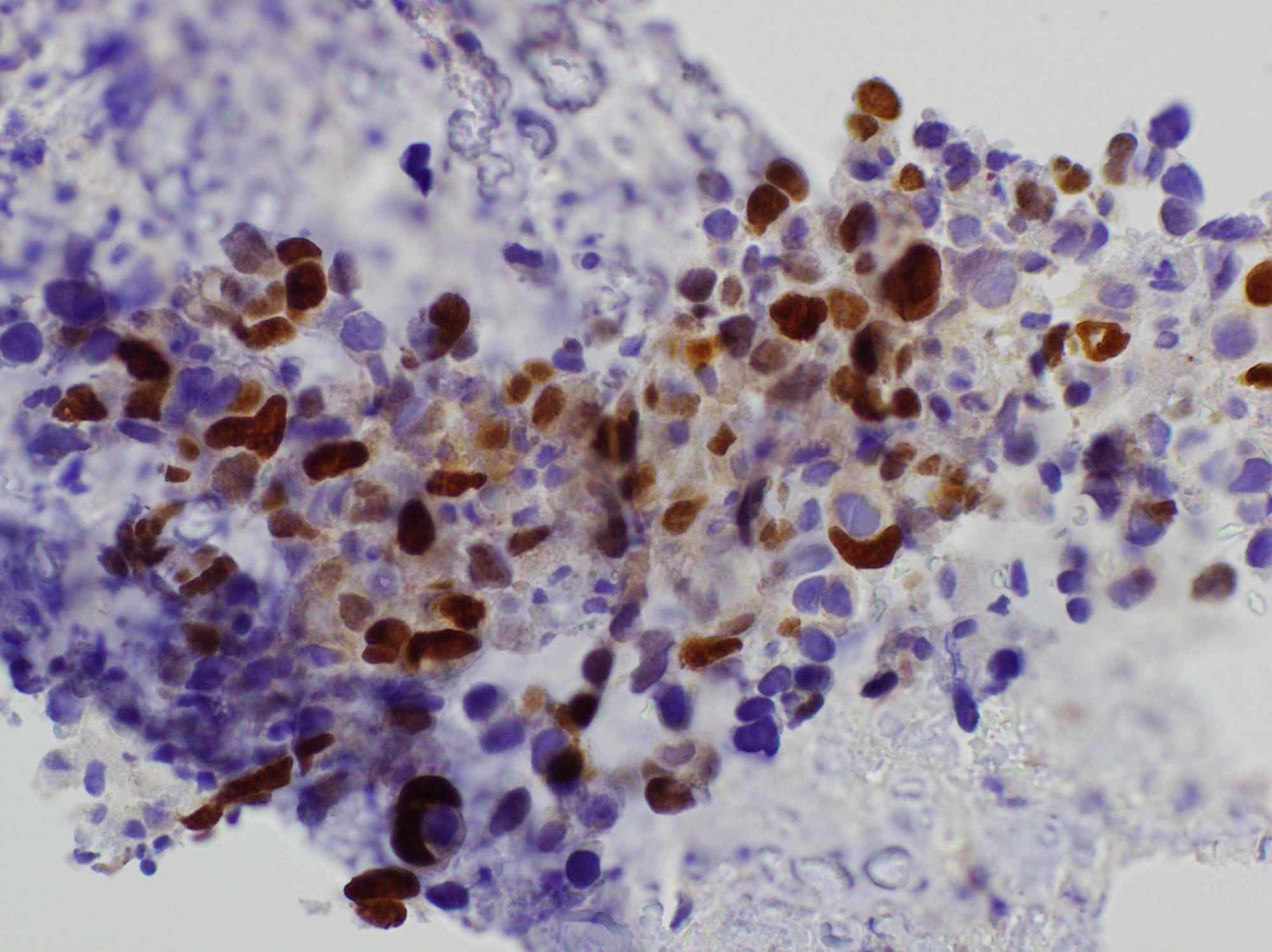
Mediastinal mass core biopsy: p 63 stain.

A transthoracic echocardiogram (TTE) was checked that showed small hyperdynamic left and right ventricle with evidence of ventricular diastolic collapse, 3 cm circumferential pericardial effusion, and excessive respirophasic variation, consistent with cardiac tamponade. An emergent pericardiocentesis and thoracentesis were done. The pericardial fluid cytology showed malignant cells. Oncology was consulted. A tissue diagnosis to elucidate the exact nature of the mediastinal mass along with the positron emission tomography (PET)/CT scan for staging was recommended. The patient was discharged and had an outpatient mediastinal mass core biopsy. It revealed high-grade non-keratinizing squamous cell TC (Figure 4). On immunohistochemistry, it stained positive for pan-cytokeratin (Figure 5), high molecular weight cytokeratin (Figure 6), CK5/6 (Figure 7), E-cadherin, p63 (Figure 8), epithelial membrane antigen (EMA), and BerEp4, and negative staining for CK7, CK20, S100, HMB45, CD3, CD20, and CD45. CD3 did demonstrate various T cells, while CD20 showed only rare B cells. CD5 appeared to demonstrate positive staining only by T cells. There was no evidence of background thymoma.

**Figure 9:**
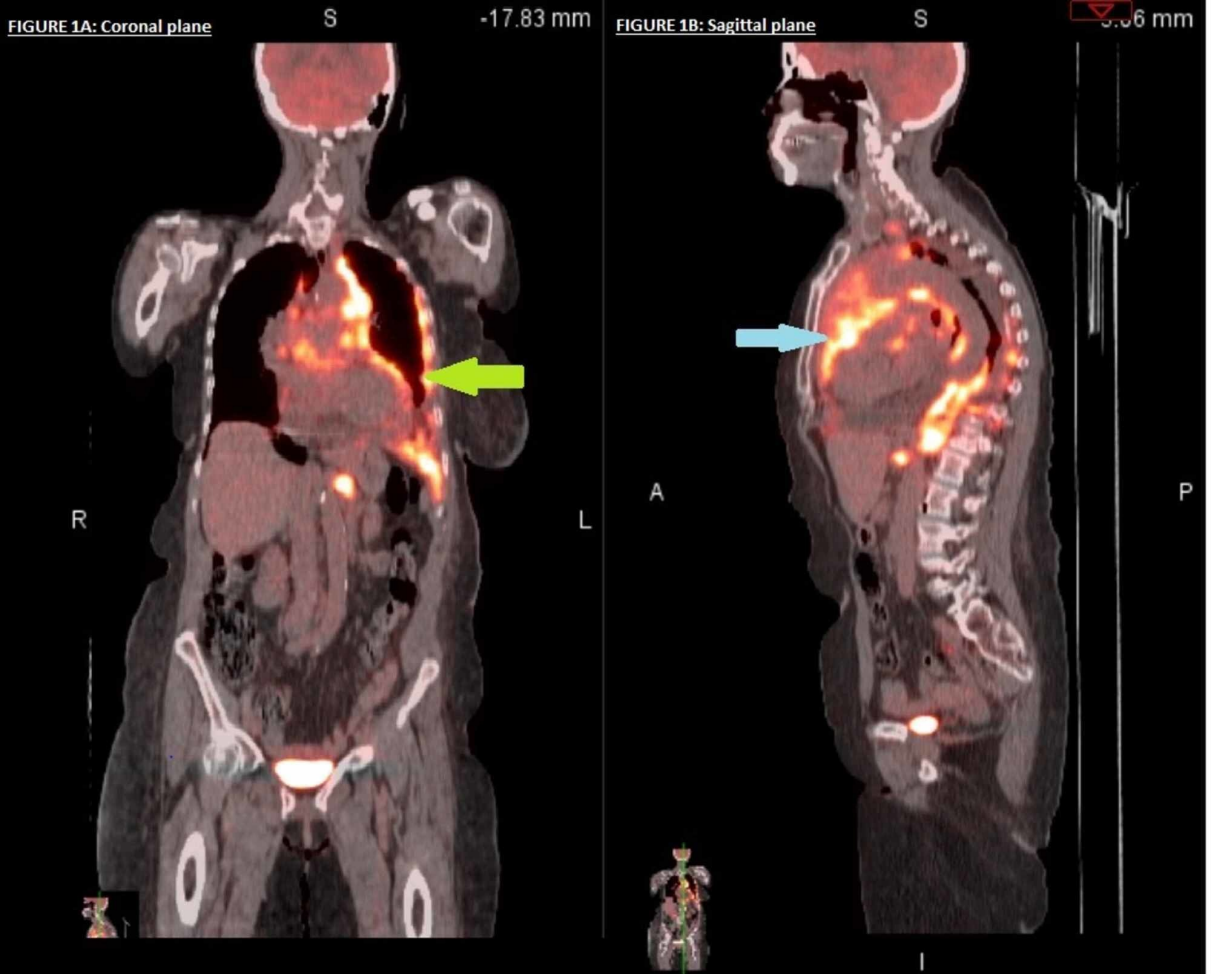
Positron emission tomography (PET)/CT showing hypermetabolic marked pleural thickening throughout the left hemithorax periphery (green arrow) and anterior mediastinal mass (blue arrow).

Flow cytometry findings indicated a non-lymphoid nature of this malignancy. In the meantime, the patient was readmitted to the hospital with symptoms of worsening dyspnea, secondary to recurrent malignant pericardial effusion and left pleural effusion, and underwent pericardial window and pleural effusion drainage catheter placement. A PET-CT scan showed an increased size of the hypermetabolic anterior mediastinal mass (11.5 cm transverse and 6.1 cm anteroposterior dimension), with a maximum standardized uptake value (SUV) 16.9. It also revealed a new hypermetabolic (maximum SUV 16.9), marked pleural thickening throughout the left hemithorax periphery consistent with the pleural extension of disease (Figure 9). There was evidence of osseous and lymph node (hilar, retrocrural, supraclavicular, left iliac chain, and mediastinal lymph nodes), metastatic disease, and a new moderate-sized pericardial effusion.

The patient was planned for radiation and chemotherapy. However, the patient succumbed to the disease before the therapy could be instituted. 

## Discussion

The TCs are extremely rare and comprise about 15%-20% of all the TETs [1]. Unlike thymomas, TCs tend to be more aggressive and are known for cellular atypia as well as lymphatic and hematogenous metastatic spread resulting in a poor prognosis [2]. The five-year survival rates for thymomas and TCs are approximately 78% and 40%, respectively [3]. 

World Health Organization (WHO) has given the classification of TC based on their histological appearance (Table 2) [1].

**Table 2:** WHO classification for thymic carcinoma

WHO classification
1. Squamous cell carcinoma
2. Basaloid carcinoma
3. Mucoepidermoid carcinoma
4. Lymphoepithelioma-like carcinoma
5. Sarcomatoid carcinoma
6. Clear cell carcinoma
7. Adenocarcinoma:
Papillary adenocarcinoma,
Thymic carcinoma with adenoid cystic carcinoma-like features,
Mucinous adenocarcinoma, and
Adenocarcinoma, not otherwise specified (NOS)
8. NUT (nuclear protein of the testis) carcinoma
9. Undifferentiated carcinoma
10. Other rare thymic carcinoma:
Adenosquamous carcinoma,
Hepatoid carcinoma, and
Thymic carcinoma, NOS
11. Combined thymic carcinoma

In 1977, TC was first described by Shimosato et al. in their study on thymic squamous cell carcinoma (TSCC) [4]. Histologically, squamous cell thymic cancer is characterized by large islands of polygonal cells typically in a lobular formation, nuclei are vesicular with prominent eosinophilic nucleoli, and abundant eosinophilic cytoplasm. They may be well, moderately, or poorly differentiated [1]. Immunohistochemical (IHC) studies are required to make a diagnosis, especially given the ambiguity of histology between thymomas and TCs as well as squamous cell carcinomas elsewhere in the body. Several novel IHC markers have been discovered. For TSCC, it typically includes cytokeratin (CK) 5/6, p63, CD117, CD5, GLUT-1 (glucose transporter 1), and MUC-1 (transmembrane mucin). 

TC typically presents as an anterior mediastinal mass noted incidentally on a radiographic imaging study, or as symptoms arising from compression of the nearby mediastinal structures. This can be cough, chest or neck pain, phrenic nerve palsy, and superior vena cava syndrome [5]. Pericardial effusion or cardiac tamponade as an initial presentation of this cancer is extremely rare. Especially in our case, the severity and frequent recurrence of the pericardial effusions contributed to recurrent hospitalizations and delay in the therapy of this aggressive cancer, resulting in a poor patient outcome. 

Treatment of the TCs is per National Comprehensive Cancer Network (NCCN) guidelines, and should typically involve a multidisciplinary team approach with oncology, cardiothoracic surgery, radiation oncology, and pathology doctors with expertise in the management of TCs. For surgically resectable tumor, defined as a well-defined anterior mediastinal mass in the thymic bed with no lymphadenopathy or continuity with the thyroid and negative tumor markers, a complete thymectomy with complete tumor excision is recommended. Also, since the recurrence rate is high for the TCs, it is important to give postoperative radiation therapy with or without chemotherapy, depending on the extent of resection [6]. For locally advanced and non-resectable tumors, as determined by a thoracic surgeon, a core needle biopsy is done to obtain a tissue diagnosis. This should be followed by definitive radiation and chemotherapy. A preferred first-line combination chemotherapy regimen consists of carboplatin and paclitaxel administered every three weeks. The second-line therapy includes sunitinib, pemetrexed, everolimus, paclitaxel, octreotide with or without prednisone, gemcitabine with or without capecitabine, 5-fluorouracil (5-FU), etoposide, ifosfamide, and pembrolizumab [7,8]. However, there is very minimal evidence on the effectiveness of second-line therapies. 

The survival rates for TCs depend on the stage of cancer and the resectability. Survival for patients with a completely resectable tumor is longer than the ones who have a non-resectable tumor or have an incomplete resection of the tumor [6]. 

## Conclusions

Primary squamous TC presenting as cardiac tamponade and recurrent pericardial effusion is a rare phenomenon. As seen in our patient, despite having a diagnosis, the recurrent hospitalization for cardiac tamponade and pleural effusion lead to an overall delay in the therapy of this aggressive malignancy. TCs are morphologically and pathologically distinct from the thymomas in lacking the immature T cells and having the cytoarchitectural features similar to squamous cell carcinoma elsewhere. Given the highly aggressive course, early diagnosis and prompt treatment are of utmost importance. The prognosis is reported to be extremely poor if there is a delay in the administration of chemo or irradiation therapy. 
